# Knowledge, attitude, and practice towards e-cigarettes among adults in the Kingdom of Saudi Arabia: a cross-sectional study

**DOI:** 10.3389/fpubh.2025.1701571

**Published:** 2025-11-11

**Authors:** Rahaf Saad Alhemayed, Mohammed Khaled Al-Hanawi

**Affiliations:** 1 Department of Health Services and Hospitals Administration, Faculty of Economics and Administration, King Abdulaziz University, Jeddah, Saudi Arabia; 2 Health Economics Research Group, King Abdulaziz University, Jeddah, Saudi Arabia

**Keywords:** attitudes, e-cigarette, health risks, knowledge, practice, Saudi Arabia

## Abstract

**Background:**

Amid increasing evidence of the health risks linked to traditional combustible cigarettes, electronic cigarettes (e-cigarettes) have become widely adopted. However, the rise in e-cigarette use also poses emerging health risks. This study examines the knowledge, attitudes, and practices (KAP) related to e-cigarette use among the general population in Saudi Arabia.

**Methods:**

This cross-sectional study collected data through a structured, self-administered online questionnaire from 1,233 participants during the period from 10 April 2025 to 4 May 2025. Descriptive statistics, including frequencies and percentages, were used to summarise participants’ demographic and socio-economic characteristics. Chi-square (*χ*^2^) tests were used to examine the bivariate associations between socio-demographic variables and each KAP domain. Additionally, multivariable logistic regression analyses were performed to examine factors associated with sufficient knowledge, positive attitudes, and positive practices regarding e-cigarette use.

**Results:**

The mean knowledge score was 9.50 (SD = 3.49, range: 0–15), with approximately two-thirds of the sample (*n* = 819) classified as having sufficient knowledge. The mean attitude score was 23.96 (SD = 6.68, range: 9–35), with 59% of the sample (*n* = 731) exhibiting a positive attitude. The mean practice score was 1.56 (SD = 1.09, range: 0–3), with less than half the sample demonstrating positive practice. Participants with sufficient knowledge were over twice as likely to hold a positive attitude [adjusted odds ratio (AOR) = 2.156; 95% confidence interval (CI): 1.509–3.080; *p* < 0.01], while both sufficient knowledge (AOR = 2.195; 95% CI: 1.343–3.589; *p* < 0.01) and a positive attitude (AOR = 13.842; 95% CI: 8.190–23.396; *p* < 0.01) strongly associated with positive practices.

**Conclusion:**

This study demonstrated positive correlations among knowledge, attitude, and practice of e-cigarettes, emphasising that tailored, nationwide awareness campaigns addressing diverse socio-demographic profiles can improve knowledge, modify attitudes, and ultimately reduce e-cigarette use.

## Introduction

In response to increasing evidence of the health risks associated with traditional combustible cigarettes, there has been a trend in shifting towards electronic cigarette (e-cigarette) use (1). E-cigarettes, also known as “vape pens” or “electronic nicotine delivery systems”, are battery-powered devices that aerosolise a liquid typically composed of propylene glycol, vegetable glycerine, nicotine, flavourings, and other chemicals (2, 3). Marketed as a less harmful alternative to traditional smoking, these devices have also been promoted as potential aids for smoking cessation. Although e-cigarettes are widely perceived as safer alternatives to conventional tobacco products, their long-term health effects remain uncertain and continue to be the focus of scientific investigation (4). Nonetheless, their accessibility, wide range of appealing flavours, and targeted promotion, particularly through social media, have contributed significantly to their popularity (5).

In recent years, global sales of e-cigarettes have escalated dramatically, with usage disproportionately concentrated among adolescents and young adults (2). Between 2011 and 2021, the global number of e-cigarette users rose from approximately 7 million to over 82 million (6). This rapid proliferation has triggered growing public health concerns, particularly in light of increasing uptake among younger populations (3, 7, 8). In the United States, e-cigarette use among high school students surged by 78% between 2017 and 2018 alone (9), with an overall increase of more than 19% observed between 2011 and 2018 (10). Comparable trends have been documented in other high-income settings, including Canada and the United Kingdom (11). However, emerging data also demonstrate substantial growth among adult populations. Among the adult population, e-cigarette use also exhibits significant geographic variation. In 2015, the prevalence was estimated at 0.2% in Vietnam and 2.3% in the Philippines, rising to 18.2% in the Russian Federation by 2016 (12). In Europe, more than 8.3 million adults across 12 countries were reported to be e-cigarette users (13). Globally, between 2015 and 2018, approximately 18.3 million adults in 14 countries used e-cigarettes (14), contributing to an estimated 58.1 million e-cigarette users worldwide in 2018 (15).

Multiple factors contribute to the rising use of e-cigarettes, including perceived usefulness in quitting smoking, lower prices, and enhanced sensory appeal such as better taste and smell compared to those of traditional cigarettes (16, 17). Some studies suggest that e-cigarettes may support smoking reduction, with users reporting decreased consumption of combustible tobacco, reduced nicotine dependence, and greater motivation to quit (18). Despite widespread awareness, many misconceptions persist regarding the safety and addictive potential of e-cigarettes (19). For instance, while 74.3% of dental students were aware of e-cigarettes, their understanding of nicotine content remained poor, indicating widespread misinformation about product composition (20). Furthermore, smokers who used e-cigarettes as a smoking cessation aid were, counterintuitively, less likely to succeed in stopping smoking (21). Additionally, e-cigarette usage has increased among adolescents and young adults with no prior smoking history (11), and evidence shows that young e-cigarette users are 3.6 times more likely to eventually initiate smoking combustible cigarettes (22). Recent global evidence also indicates a rise in dual use among adults—those who smoke conventional cigarettes while also regularly using e-cigarettes—which further complicates cessation efforts and public health messaging (14).

While some researchers argue that e-cigarettes are less harmful because they lack combustion and emit fewer toxicants (23), their increasing global use remains a significant health concern (24). Studies have linked e-cigarettes to adverse outcomes such as thermal injuries, airway obstruction, myocardial infarction, and vascular endothelial dysfunction (25–27), along with respiratory tract irritation, palpitations, elevated blood pressure, and an increased risk of tumours among nonusers exposed to second-hand e-cigarette emissions (28). Consequently, numerous public health authorities now classify e-cigarette use as a serious and growing threat to public health (29). While some of the more typical side effects—such as nausea, coughing, and inflammation—may appear mild, vaping can also have more serious consequences, such as negative effects on the central nervous system (30, 31).

Persistent knowledge gaps about e-cigarettes underscore the need for additional empirical research on the topic. As the use of these devices continues to grow, alongside increasing evidence of health risks, addressing misconceptions has become a public health priority. The Kingdom of Saudi Arabia (KSA) is a key example of a country with increasing prevalence of e-cigarette use. Significantly higher usage rates have been observed in specific groups, with a prevalence of 27.7% among university students (32). Among adults, 26.3% reported ever using e-cigarettes (33), and among 325 adult male smokers, one-third (33.5%) are current users (34). However, as noted by Al-Hamdani and Hopkins (35), the overreliance on university-based samples and the significant variability in current data highlight the need for further research to fill critical knowledge gaps.

In particular, previous studies on e-cigarette use in the KSA have targeted first-year university students (36), general undergraduate groups (37), and medical students across various institutions (32, 38, 39). Although youth—particularly those involved in the medical sector—represents a significant demographic, e-cigarette use reaches far beyond academic and healthcare settings, extending into the broader population. However, nationally representative studies on adult populations remain limited, particularly those exploring knowledge, attitudes, and practices (KAP) related to e-cigarettes and their associated socio-demographic and behavioural factors. Therefore, there is a need to update and expand the current available evidence with more comprehensive data from the general public. Saudi Arabia presents a compelling case given that the healthcare system is already under strain and rising e-cigarette use could worsen the burden of non-communicable diseases (40, 41). To address these gaps, the aim of this study was to assess the KAP related to e-cigarettes among adults in the general population of the KSA. By broadening and deepening the existing evidence base, this study contributes to national efforts to inform public health interventions and tobacco control policies consistent with Saudi Vision 2030.

## Materials and methods

### Study design and sample

This cross-sectional study was conducted to assess the KAP towards e-cigarettes among adults in Saudi Arabia. Data were collected online from 10 April 2025 to 4 May 2025 via a structured, self-administered online questionnaire, using SurveyMonkey. Considering the widespread use of the internet and social media platforms in the KSA (42), the survey link was distributed via commonly used channels such as Twitter and WhatsApp. A snowball sampling approach was employed, whereby initial participants were invited to complete the survey and subsequently encouraged to share the link with their personal and professional networks to enhance participant reach.

To enhance the study’s generalizability and strengthen the external validity of the findings, efforts were made to reach a broad and diverse sample of participants. According to the most recent 2024 national census by the General Authority for Statistics in Saudi Arabia, the total population is approximately 35.3 million individuals (43). The minimum required sample size was calculated using an online sample size calculator (44). Based on a 99% confidence level, a 5% margin of error, a 50% response distribution, and the total population size, the recommended sample size was determined to be 664 participants.

### Study instrument

The self-administered questionnaire was designed and developed based on similar studies to assess KAP towards e-cigarettes (16, 31). The questionnaire was initially designed in English and was then translated into Arabic. One author (RA) translated the questionnaire from English to Arabic, which was then back-translated to English by MA-H to ensure the accuracy of meaning. The Arabic version of the questionnaire was used to collect the data.

The questionnaire consisted of four main parts. The first part of the questionnaire collected general information and information on respondents’ socio-economic and demographic characteristics. The second part of the questionnaire assessed participants’ knowledge of e-cigarettes. The third part of the questionnaire assessed participants’ attitudes towards e-cigarettes using seven statements. The final part of the questionnaire assessed participants’ practices related to e-cigarettes.

Before accessing the questionnaire, participants were provided with a clear overview of the study’s objectives and were informed of their right to withdraw from participation at any time without the need to provide justification. They were assured that their responses would remain anonymous and treated with strict confidentiality. Eligibility criteria included being a resident of Saudi Arabia, at least 18 years of age, capable of understanding the questionnaire content, and providing informed consent. Only those who confirmed having heard of e-cigarettes were allowed to proceed. Informed consent was obtained electronically prior to initiating the survey.

### Outcome variables

Knowledge about e-cigarettes was assessed using 15 items covering topics such as regulatory approval, potential toxicity, carcinogenic risks, addictive properties, effects on health (including cardiovascular, pulmonary, foetal development, and second-hand exposure), the presence of harmful chemicals and nicotine, and misconceptions about the safety of e-cigarette use among vulnerable populations (e.g., children and pregnant women). Respondents were asked to respond to knowledge items as either true or false, with an additional “do not know” option. Participants received one point for each correct answer, while incorrect or uncertain responses (e.g., “do not know”) were scored as zero. Total knowledge scores ranged from 0 to 15, with higher scores reflecting greater knowledge. The reliability of the knowledge items was evaluated using Cronbach’s alpha, which yielded a coefficient of 0.803, indicating good internal consistency (45).

Attitudes towards e-cigarettes were measured using seven statements related to perceptions of social acceptability, public use, government regulation, social image, and whether e-cigarette users should be regarded as non-smokers or encouraged to use e-cigarettes as a safer alternative. Responses were rated on a five-point Likert scale (strongly disagree, disagree, neutral, agree, strongly agree) to yield a total attitude score ranging from 7 to 35, with higher scores reflecting more positive attitudes. The reliability of the attitude items was assessed using Cronbach’s alpha, which produced a coefficient of 0.852, indicating a good level of internal consistency.

Practices related to e-cigarette use were assessed using three items on lifetime and current use of e-cigarettes, as well as willingness to promote or recommend their use to others. Each response reflecting a favourable or healthy behaviour was scored as one, while responses indicating unfavourable or risky behaviour were scored as zero. Total practice scores ranged from 0 to 3, with higher scores indicating more positive practices.

The scores for the knowledge and attitude scales were then dichotomized into binary variables. A cut-off score of ≥9 was used to categorise participants as having sufficient knowledge, corresponding to 60% or more correct responses. Similarly, a cut-off score of ≥21 (equivalent to 60% of the maximum possible score) was used to define a positive attitude, and a cut-off score of ≥2 was applied to classify participants as having positive practice. This percentage cut-off point was applied following other studies assessing knowledge, attitudes, and behaviour towards e-cigarettes (31).

### Exposure variables

General information, along with socio-economic and demographic characteristics—including age, sex, marital status, educational attainment, employment status, nationality, monthly income, region of residence, and smoking status—were included as exposure variables. Age was categorised into six groups: 18–24 years (reference category), 25–34, 35–44, 45–54, 55–64, and ≥65 years. Sex was coded as a binary variable, with males assigned a value of 1 and females a value of 0. Marital status was also binary-coded, with married individuals coded as 1 and unmarried individuals (including those who were divorced, widowed, or never married) coded as 0.

Educational attainment was grouped into three categories: secondary school or below (reference category), university degree, and postgraduate degree. Employment status was categorised into six groups: unemployed (reference category), government employee, non-government employee, self-employed, retiree, and student. Nationality was coded as 1 for Saudi participants and 0 for non-Saudis. Monthly income (in Saudi Riyals [SR]; 1 SR ≈ USD 0.27) was classified into five brackets: <SR 5,000 (reference category), SR 5,000 to <10,000, SR 10,000 to <15,000, SR 15,000 to <20,000, and ≥SR 20,000.

Region of residence was classified into five broader geographic zones based on the 13 administrative regions of the KSA: Central (Riyadh; reference category), Western (Makkah, Madinah, and Al-Baha), Northern (Northern Borders, Al-Jouf, Hail, and Tabuk), Southern (Aseer, Jazan, and Najran), and Eastern (Eastern Province and Qassim). Smoking status was dichotomized, with current smokers of any tobacco product (e.g., cigarettes, shisha, cigars) coded as 1, and non-smokers as 0.

### Statistical analysis

The study employed univariate, bivariate, and multivariable regression analyses. Univariate analyses were conducted to describe demographic and socioeconomic characteristics of the participants using frequencies and percentages. Bivariate associations between socio-demographic variables and each of the KAP domains were examined using Chi-square (*χ*^2^) tests. To identify factors associated with sufficient knowledge, positive attitude, and positive practice towards e-cigarettes, multivariable logistic regression analyses were performed. Adjusted odds ratios (AORs) with corresponding 95% confidence intervals (CIs) were calculated. All statistical analyses were conducted using STATA version 16.1 (StataCorp LLC, College Station, TX, USA).

## Results

### Socio-economic and demographic characteristics

A total of 1,282 individuals initially participated in the study. After excluding incomplete or invalid responses, the final analytical sample consisted of 1,233 participants. Table 1 shows the socio-economic and demographic characteristics of the study participants.

**Table 1:** Socio-economic and demographic characteristics of the study participants (*n* = 1,233).

Variable	*N*	%
Age		
18–24	160	12.98
25–34	491	39.82
35–44	318	25.79
45–54	138	11.19
55–64	76	6.16
+65	50	4.06
Sex		
Female	267	21.65
Male	966	78.35
Marital status		
Unmarried	448	36.33
Married	785	63.67
Educational attainment		
Secondary school or below	508	41.20
University degree	567	45.99
Postgraduate degree	158	12.81
Employment status		
Unemployed	136	11.03
Government employee	419	33.98
Non-government employee	275	22.30
Self-employed	162	13.14
Retiree	116	9.41
Student	125	10.14
Nationality		
Non-Saudi	63	5.11
Saudi	1,170	94.89
Monthly income		
<5000	424	34.39
5,000 to <10,000	415	33.66
10,000 to <15,000	153	12.41
15,000 to <20,000	115	9.33
≥20,000	126	10.22
Region		
Central	345	27.98
Western	491	39.82
Northern	139	11.27
Southern	105	8.52
Eastern	153	12.41
Smoking status		
No	495	40.15
Yes	738	59.85
Total	1,233	

As shown in Table 1, participants were predominantly in their mid-adulthood, with the largest proportion aged 25–34 years. The majority were male, married, held at least a university degree and were Saudi. Employment was diverse, though over one-third were government employees. While income levels varied, approximately two-thirds of the sample earned below 10,000 SR per month. Geographically, participants were most commonly from the Western and Central regions. Notably, nearly 60% of participants reported being current smokers.

The mean knowledge score was 9.50 (SD = 3.49; range 0–15), with approximately two-thirds (*n* = 819, 66.42%) of participants classified as having sufficient knowledge about e-cigarettes. The mean attitude score was 23.96 (SD = 6.68; range: 9–35), with just under 60% (*n* = 731, 59.29%) reported a positive attitude. The mean practice score was 1.56 (SD = 1.09; range: 0–3), with less than half (*n* = 545, 44.2%) demonstrated positive practice.

### Bivariate analysis of KAP towards e-cigarettes

Chi-square tests demonstrated significant associations between knowledge scores and all key socio-demographic variables except nationality and income (Table 2). Knowledge levels varied significantly by age group, sex, marital status, educational attainment, employment status, region, and smoking status (*p* < 0.01). Notably, females, married individuals, those with higher education, and non-smokers were more likely to demonstrate sufficient knowledge.

**Table 2:** Associations of socio-demographic characteristics with knowledge, attitude, and practice towards e-cigarettes.

Variable	Knowledge					Attitude					Practice				
	Poor		Sufficient		Chi-square	Negative		Positive		Chi-square	Negative		Positive		Chi-square
	*N*	%	*N*	%		*N*	%	*N*	%		*N*	%	*N*	%	
Age					56.471^***^					16.504^***^					41.299^***^
18–24	70	43.75	90	56.25		61	38.13	99	61.88		78	48.75	82	51.25	
25–34	123	25.05	368	74.95		212	43.18	279	56.82		306	62.32	185	37.68	
35–44	98	30.82	220	69.18		147	46.23	171	53.77		195	61.32	123	38.68	
45–54	54	39.13	84	60.87		46	33.33	92	66.67		64	46.38	74	53.62	
55–64	36	47.37	40	52.63		22	28.95	54	71.05		28	36.84	48	63.16	
+65	33	66.00	17	34.00		14	28.00	36	72.00		17	34.00	33	66.00	
Sex					57.181^***^					85.503^***^					106.091^***^
Female	38	14.23	229	85.77		43	16.10	224	83.90		75	28.09	192	71.91	
Male	376	38.92	590	61.08		459	47.52	507	52.48		613	63.46	353	63.54	
Marital status					12.837^***^					1.283					0.225
Unmarried	179	39.96	269	60.04		173	38.62	275	61.38		246	54.91	202	45.09	
Married	235	29.94	550	70.06		329	41.91	456	58.09		442	56.31	343	43.69	
Educational attainment					73.795^***^					75.325^***^					135.79^***^
Secondary school or below	237	46.65	271	53.35		272	53.54	236	46.46		377	74.21	131	25.79	
University degree	124	21.87	443	78.13		202	35.63	365	64.37		266	46.91	301	53.09	
Postgraduate degree	53	33.54	105	66.46		28	17.72	130	82.28		45	28.48	113	71.52	
Employment status					90.258^***^					43.514^***^					64.280^***^
Unemployed	40	29.41	96	70.59		29	21.32	107	78.68		48	35.29	88	64.71	
Government employee	87	20.76	332	79.24		172	41.05	247	58.95		231	55.13	188	44.87	
Non–government employee	84	30.55	191	69.45		133	48.63	142	51.64		182	66.18	93	33.82	
Self–employed	88	54.32	74	45.68		85	52.47	77	47.53		115	70.99	47	29.01	
Retiree	63	54.31	53	45.69		34	29.31	82	70.69		45	38.79	71	61.21	
Student	52	41.60	73	58.40		49	39.20	76	60.80		67	53.60	58	46.40	
Nationality					1.992					0.487					17.697^***^
Non–Saudi	16	25.40	47	74.60		23	36.51	40	63.49		19	30.16	44	69.84	
Saudi	398	34.02	772	65.98		479	40.94	691	59.06		669	57.18	501	42.82	
Monthly income (SR)					4.104					110.554^***^					100.698^***^
<5000	150	35.38	274	64.62		161	37.97	263	62.03		219	51.65	205	48.35	
5,000 to <10,000	124	29.88	291	70.12		242	58.31	173	41.69		305	73.49	110	26.51	
10,000 to <15,000	52	33.99	101	66.01		57	37.25	96	62.75		79	51.63	74	48.37	
15,000 to <20,000	42	36.52	73	63.48		24	20.87	91	79.31		47	40.87	68	59.13	
≥20,000	46	36.51	80	63.49		18	14.29	108	85.71		38	30.16	88	69.84	
Region					172.944^***^					127.857^***^					71.964^***^
Central	204	59.13	141	40.87		131	37.97	214	62.03		183	53.04	162	46.96	
Western	111	22.61	380	77.39		133	27.09	358	72.91		224	45.62	267	54.38	
Northern	10	7.19	129	92.81		108	77.70	31	22.30		113	81.29	26	18.71	
Southern	31	29.52	74	70.48		51	48.57	54	51.43		60	57.14	45	42.86	
Eastern	58	37.91	95	62.09		79	51.63	74	48.37		108	70.59	45	29.41	
Smoking status					39.679^***^					351.435^***^					712.673^***^
No	115	23.23	380	76.77		43	8.69	452	91.31		48	9.70	447	90.30	
Yes	299	40.51	439	59.49		459	62.20	279	37.80		640	86.72	98	13.28	

^***^p < 0.01.

Attitude towards e-cigarettes was significantly associated with most socio-demographic variables except marital status and nationality. Positive attitudes were more common among females, individuals with higher education levels, higher income brackets, and those residing in the Western region. Among the socio-demographic variables, smoking status showed the strongest association with attitude, in which non-smokers overwhelmingly reported more positive attitudes (*p* < 0.01). Practice towards e-cigarettes also exhibited significant associations with nearly all variables except marital status. Positive practices were more frequently reported by females, those with higher education, higher income, and non-smokers. Nationality was also significantly associated with practice (*p* < 0.01), with non-Saudis more likely to report positive behaviours. Regional differences were also prominent, particularly with lower positive practice scores evident in respondents from the Northern and Eastern regions.

### Multivariable analysis of factors associated with KAP towards e-cigarettes

Table 3 shows the results of the multivariable logistic regression analysis to identify factors associated with participants’ KAP towards e-cigarettes. Several variables were significantly associated with sufficient knowledge. Females, married participants, those with a university degree, and government employees were more likely to possess sufficient knowledge compared to their respective counterparts. Regionally, participants from the Northern, Western, Southern, and Eastern regions had significantly higher odds of sufficient knowledge compared to those in the Central region. Smokers were significantly less likely to have sufficient knowledge than non-smokers.

**Table 3:** Logistic regression of factors associated with knowledge, attitude, and practice towards e-cigarette.

Variables	Knowledge		Attitude		Practice	
	AOR	95% CI	AOR	95% CI	AOR	95% CI
Age						
18–24	Reference		Reference		Reference	
25–34	1.631	(0.833–3.196)	1.529	(0.774–3.022)	0.418	(0.172–1.018)
35–44	1.174	(0.561–2.458)	1.652	(0.789–3.462)	0.648	(0.237–1.770)
45–54	0.800	(0.358–1.786)	2.069	(0.914–4.686)	1.215	(0.405–3.646)
55–64	0.662	(0.255–1.715)	1.073	(0.387–2.979)	1.252	(0.327–4.791)
+65	0.473	(0.153–1.465)	1.011	(0.317–2.848)	1.789	(0.326–9.799)
Sex						
Female	Reference		Reference		Reference	
Male	0.308^***^	(0.198–0.479)	0.629^**^	(0.398–0.993)	0.981	(0.558–1.724)
Marital status						
Unmarried	Reference		Reference		Reference	
Married	1.647^***^	(1.147–2.364)	1.011	(0.725–1.410)	1.183	(0.710–1.970)
Educational attainment						
Secondary school or below	Reference		Reference		Reference	
University degree	2.123^***^	(1.498–3.010)	1.157	(0.816–1.640)	2.423^***^	(1.465–4.007)
Postgraduate degree	1.222	(0.712–2.099)	1.515	(0.826–2.777)	4.296^***^	(1.958–9.430)
Employment status						
Unemployed	Reference		Reference		Reference	
Government employee	2.017^**^	(1.075–3.782)	0.375^***^	(0.195–0.719)	0.867	(0.357–2.110)
Non–government employee	1.426	(0.788–2.580)	0.416^***^	(0.222–0.777)	0.618	(0.266–1.438)
Self–employed	0.516^**^	(0.271–0.980)	0.428^**^	(0.218–0.840)	0.668	(0.257–1.736)
Retiree	1.281	(0.581–2.825)	0.590	(0.249–1.395)	0.968	(0.276–3.394)
Student	1.730	(0.790–3.791)	0.643	(0.285–1.451)	0.346^**^	(0.121–0.988)
Nationality						
Non–Saudi	Reference		Reference		Reference	
Saudi	1.142	(0.585–2.229)	1.748	(0.856–3.570)	0.227^***^	(0.092–0.559)
Monthly income (Saudi Riyal)						
<5000	Reference		Reference		Reference	
5,000 to <10,000	1.143	(0.757–1.725)	0.822	(0.546–1.239)	0.707	(0.377–1.326)
10,000 to <15,000	0.716	(0.409–1.253)	1.681	(0.955–2.958)	0.744	(0.333–1.661)
15,000 to <20,000	1.032	(0.543–1.961)	3.423^***^	(1.691–6.925)	0.455	(0.184–1.126)
≥20,000	1.559	(0.780–3.114)	4.606^***^	(2.097–10.116)	0.493	(0.187–1.300)
Region						
Central	Reference		Reference		Reference	
Western	4.959^***^	(3.437–7.157)	2.003^***^	(1.306–3.070)	1.821^**^	(1.022–3.246)
Northern	24.982^***^	(11.913–52.387)	0.288^***^	(0.158–0.524)	1.822	(0.785–4.231)
Southern	4.061^***^	(2.322–7.104)	1.109	(0.796–1.691)	2.29	(0.941–5.572)
Eastern	4.025^***^	(2.468–6.565)	1.368	(0.807–2.318)	1.658	(0.761–3.616)
Smoking status						
No	Reference		Reference		Reference	
Yes	0.334^***^	(0.235–0.474)	0.087^***^	(0.058–0.129)	0.026^***^	(0.016–0.043)
Knowledge						
Poor			Reference		Reference	
Satisfactory			2.156^***^	(1.509–3.080)	2.195^***^	(1.343–3.589)
Attitude						
Negative					Reference	
Positive					13.842^***^	(8.190–23.396)
Observations	1,233		1,233		1,233	
Pseudo *R*-squared	0.256		0.339		0.622	
Chi-square	402.744^***^		564.254^***^		1053.588^***^	

95% confidence intervals are in parentheses. AOR, adjusted odds ratio; CI, confidence interval; ^***^p < 0.01, ^**^p < 0.05.

Regarding attitude towards e-cigarettes, male participants had significantly lower odds of having a positive attitude compared to females. Government employees, non-government employees, and self-employed individuals were significantly less likely to have a positive attitude compared to the unemployed. Higher-income participants, especially those earning 15,000 to <20,000 SR and ≥20,000 SR, were more likely to report a positive attitude compared to those earning less than 5,000 SR. Regionally, compared to participants in the Central region, those residing in the Western region had significantly higher odds of a positive attitude, while participants from the Northern region had significantly lower odds. Smokers were substantially less likely to have a positive attitude about e-cigarettes than non-smokers. Furthermore, participants with sufficient knowledge were more than twice as likely to exhibit a positive attitude.

Educational attainment was one of the most influential factors associated with the practice towards e-cigarettes: individuals with a university or postgraduate degree were significantly more likely to demonstrate positive practice. Conversely, students were less likely to report positive practice, as were Saudi nationals compared to non-Saudis. Participants residing in the Western region had higher odds of positive practice. Most notably, sufficient knowledge and a positive attitude were the strongest factors associated with positive practice. By contrast, smokers had drastically reduced odds of positive practice, indicating a significant barrier to behavioural change.

Several factors demonstrated consistent associations across all three KAP domains. Smoking status emerged as the most prominent factor, with current smokers significantly less likely to exhibit sufficient knowledge, hold positive attitudes, or report positive practices related to e-cigarettes. Male participants also exhibited a similar pattern, showing a reduced likelihood of having both sufficient knowledge and positive attitudes compared to females. Educational attainment showed a consistent positive influence, particularly among university degree holders, who were more likely to demonstrate both sufficient knowledge and positive practices. Regionally, participants from the Northern region exhibited markedly higher knowledge but had significantly lower attitude scores—suggesting a disconnect between awareness and behavioural change. Conversely, individuals from the Western region showed favourable outcomes in knowledge, attitude, and practice, whereas those from the Eastern region had higher knowledge but no significant difference in attitudes or behaviour compared to the Central region. These cross-domain findings underscore the need for integrated health promotion strategies that address not only awareness but also behavioural intent and cultural perceptions.

## Discussion

Amid rising concerns about public health related to e-cigarette usage, this study examined the KAP surrounding e-cigarettes among adults in the KSA. Overall, the results showed that participants with sufficient knowledge were more than twice as likely to hold positive attitudes, with both knowledge and attitude emerging as most strongly associated with positive practices. Although the ability to compare the findings to those of related studies is limited due to varied target populations, some similarities can be observed. For example, the present findings are consistent with those from Lebanon, where increased knowledge was associated with negative perceptions of e-cigarettes, suggesting a more health-conscious attitude of the population (16). Likewise, Alsanea et al. (46) emphasised that insufficient knowledge and widespread misconceptions among male college students in Saudi Arabia contributed to negative attitudes, which could pose risks to public health. These similar patterns suggest that improving knowledge may enhance public perception and reduce the use of e-cigarettes. Nevertheless, strategies should go beyond awareness campaigns to actively influence attitude formation and behavioural change.

The present study identified smoking status as the most significant factor associated with the KAP related to e-cigarettes. Current smokers were significantly less likely to exhibit sufficient knowledge, hold positive attitudes, or report positive practices than non-smokers, suggesting a substantial barrier to behavioural change. These findings align with prior research indicating that smokers consistently display lower levels of knowledge about the risks and regulatory status of e-cigarettes (46), possibly due to misinformation or avoidance of health-related truths. Smokers have also been shown to perceive e-cigarettes as more addictive (47), yet report more favourable attitudes towards their use, likely as a means of rationalising continued consumption (16). While some studies report high knowledge scores among smokers (48), this has not translated into more positive attitudes or healthier practices—findings echoed in the current study. In contrast, non-smokers not only demonstrate greater awareness but also express stronger beliefs in the harmfulness and addictiveness of e-cigarettes (36), as well as in the role of healthcare professionals in smoking prevention (49). Collectively, these findings reinforce the notion that among smokers, knowledge alone may be insufficient to drive attitude or behaviour change, highlighting the need for targeted, behaviourally informed interventions.

This study further identified significant gender disparities in knowledge and attitudes related to e-cigarettes. Male participants were significantly less likely than females to possess sufficient knowledge and demonstrated significantly lower odds of holding positive attitudes towards e-cigarettes. While some studies reported no significant gender differences in KAP towards e-cigarettes (50, 51), the current findings are consistent with other research highlighting more positive perceptions and behaviours among females. For example, Alduraywish et al. (36) found that while men perceived e-cigarettes as a smoking cessation aid, women were more likely to disapprove of their use. Similarly, Doumi et al. (31) noted that males were more accepting of e-cigarette use compared to their female counterparts. Research involving college students in India (19) and students in Palestine (52) consistently demonstrated that males have a higher acceptance and usage of e-cigarettes. Such patterns may reflect broader gendered differences in risk perception and health behaviours, with females typically engaging less in health-compromising behaviours (48). In the context of the KSA, these differences may also be shaped by prevailing socio-cultural norms, which discourage smoking among women, thereby contributing to a positive attitude and lower reported usage (31).

Regarding educational attainment, participants holding a university or postgraduate degree were significantly more likely to demonstrate sufficient knowledge and engage in positive practices related to e-cigarettes compared to those with secondary education or lower. Although education was not significantly associated with attitude, it showed a strong positive relationship with both knowledge and practice. These findings contrast with the results of a multi-country study among dental students in which greater e-cigarette knowledge was found among individuals with lower educational levels; the authors posited that more educated individuals might be more inclined to use e-cigarettes as a coping mechanism for stress (51). In contrast, the present study aligns with the broader assumption that higher educational attainment is generally associated with increased health literacy and more informed health behaviours (53). As such, enhancing educational infrastructure and integrating targeted health education programs may serve as effective strategies for reducing e-cigarette use and promoting healthier practices.

The present study found that participants with higher incomes were generally more likely to exhibit positive attitudes towards e-cigarettes. Notably, individuals earning ≥20,000 SAR had significantly higher odds of holding a positive attitude; however, no significant associations were observed with knowledge or practice. Alduraywish et al. (36) found a significant association between income and practice; however, this relationship was not observed in the current study. This discrepancy suggests that while income may shape attitudes and facilitate access to e-cigarettes, it does not necessarily correspond to greater awareness or healthier behaviours. This further underscores the need for targeted public health strategies that go beyond economic access, addressing both cognitive and behavioural aspects of e-cigarette use across socio-economic strata.

We further found that government employees had significantly higher odds of possessing sufficient knowledge about e-cigarettes, whereas self-employed individuals were less likely to demonstrate sufficient knowledge. However, across all employment categories, both government and non-government employees exhibited significantly lower odds of holding a positive attitude towards e-cigarettes compared to unemployed participants—an unexpected trend given their relatively higher knowledge levels. This discrepancy may be attributed to greater exposure to information and structured training within formal employment settings, which likely enhances knowledge. Conversely, the lower likelihood of positive attitudes among employed individuals may stem from the increased access to e-cigarettes being linked to higher socio-economic status, potentially normalising use and influencing perceptions (16). These findings highlight a nuanced relationship among employment status, knowledge, and attitudes, underscoring the importance of addressing both informational and contextual factors in public health interventions.

Regionally, participants from the Western, Northern, Southern, and Eastern regions had significantly higher odds of sufficient knowledge compared to those in the Central region. Notably, the Northern region exhibited the highest knowledge levels but significantly lower odds of positive attitudes and no association with positive practice, highlighting a disconnect between awareness and behavioural change. In contrast, the Western region demonstrated positive outcomes across all KAP domains, while the Eastern region showed high knowledge but no corresponding association with attitudes or behaviours. The Southern region displayed significantly higher odds of sufficient knowledge and a positive trend in practice, although there was no association with attitude. These findings align with broader international patterns, where multivariable analyses have demonstrated that the country context independently influences all three KAP domains (51). This underscores the importance of localised, culturally responsive health interventions that move beyond information dissemination to actively shape attitudes and behaviours.

This study significantly contributes to the literature by broadening the scope of e-cigarette research beyond the commonly studied populations of students and adolescents to include a more representative sample of adults. This work also enhances the geographic coverage by incorporating participants from all regions of Saudi Arabia and across several socio-demographic categories, thereby increasing the potential for generalisability relative to prior studies. These findings highlight the central role of knowledge and attitudes in shaping e-cigarette use, with clear implications for research, practice, and policy. Strengthening national surveillance systems and prioritising longitudinal studies are essential to generate more representative data and evaluate intervention effectiveness. Interventions should move beyond information dissemination to actively shape perceptions and promote healthy behaviours through targeted health promotion campaigns, particularly among youth and other high-risk groups. At the policy level, integrating evidence-based education with robust regulatory measures—such as stricter advertising controls, product distribution regulations, and targeted taxation—could reduce accessibility and appeal, curb initiation, and support cessation efforts.

However, the study has some limitations that should be considered when interpreting the findings. First, the use of an online snowball sampling method may have introduced selection bias, as participation relied on internet access, social media engagement, and peer referral. This approach may have led to overrepresentation of certain groups and underrepresentation of others, thereby limiting the generalizability of the findings to the entire Saudi adult population. Future studies should consider strategies to enhance representativeness and ensure more balanced inclusion across key demographic and behaviour groups. Second, the self-reported nature of the data might have been subject to recall and social desirability biases, particularly in questions regarding smoking and vaping behaviour. Third, due to the cross-sectional nature of the study, it is not possible to establish causal relationships among knowledge, attitudes, and practices. Future research can explore strategies to address these limitations and should prioritise longitudinal or experimental designs to better assess the role of e-cigarettes in smoking cessation and understand the temporal dynamics of behavioural change.

## Conclusion

With rising public health concerns over e-cigarette use, this study provides a timely and in-depth examination of the knowledge, attitudes, and practices related to e-cigarettes among adults in Saudi Arabia. The study demonstrated positive correlations among knowledge, attitude, and practice, emphasising the importance of correcting persistent misconceptions, especially about the health risks of e-cigarettes in Saudi Arabia. Therefore, comprehensive educational efforts are crucial. These should be complemented by consistent enforcement of regulations and clear public communication on e-cigarette legislation. Tailored, nationwide awareness campaigns that account for diverse socio-demographic profiles can enhance knowledge, shift attitudes, and ultimately reduce e-cigarette use. The findings underscore the crucial role of evidence-based policies and culturally responsive strategies in fostering informed decision-making and supporting cessation efforts.

## Data Availability

The datasets generated and/or analysed during the current study are not publicly available due to privacy, confidentiality, and other restrictions, but are available from the corresponding author on reasonable request.
